# Survival prediction of gastric cancer patients by Artificial Neural Network model 

**Published:** 2018

**Authors:** Jamshid Yazdani Charati, Ghasem Janbabaei, Nadia Alipour, Soraya Mohammadi, Somayeh Ghorbani Gholiabad, Afsaneh Fendereski

**Affiliations:** 1 *Department of Biostatistics, Health Sciences Research Center, Faculty of Health, Mazandaran University of Medical Sciences, Sari, Iran*; 2 *School of Medicine, Mazandaran University of Medical Sciences, Sari, Iran*; 3 *Student Research Committee, Faculty of Health, Mazandaran University of Medical Sciences, Sari, Iran*; 4 *Faculty of Health, Hemedan University of Medical Sciences, Hemedan, Iran *

**Keywords:** Gastric cancer, Survival analysis, Artificial neural network

## Abstract

**Aim:**

This study aims to predict survival rate of gastric cancer patients and identify the effective factors related to it, using artificial neural network model.

**Background:**

Gastric cancer is the most deadly disease in north and northeast provinces of Iran. A total of 430 patients with gastric cancer who referred to Baghban clinic in Sari, from early November 2006 to late October 2013 were followed.

**Methods:**

A historical cohort of patients who referred to Baghban Clinic, the cancer research center of Mazandaran University of Medical Sciences in Sari, from early November 2006 to late October 2013 was studied. Three groups of variables (demographic, biological and socio-economic) were studied. Survival rate and effective factors on survival time were calculated using Kaplan-Meier methods and artificial neural networks and the best network structure were chosen using the mean square error and ROC curve. All analyses were performed using SPSS v.18.0 and the level of significance was selected α=0.05.

**Results:**

In this research, the median survival time was 19±2.04 months. The 1 to 5-year survival rates for patients were 0.64, 0.44, 0.34, 0.24 and 0.19, respectively. The percentage of right predictions of the selected network and the area under the ROC curve were 92% and 94%, respectively. According to the results, the type of treatment, metastasis, stage of disease, histology grade, histology type and the age of diagnosis were effective factors on survival period.

**Conclusion:**

the 5 years survival rate of gastric cancer patients in Mazandaran is lower than other provinces which could be due to the delay in diagnosis or patient’s referral. Therefore, the use of screening methods and early diagnosis could be influential for improving survival rate of these patients.

## Introduction

 Cancer is caused by the uncontrollable growth of cells that is regarded as the second most common cause of death in developed countries and the third in developing ones (1). Among cancers, gastric cancer with the mortality rate of 15.5% turned out to be the deadliest cancer in 2012 (2). This type of cancer with the incidence rate of 11.4% is known as the second most common cancer in Iran (2). Also the northern and northwestern regions are regarded as high risk areas for gastric cancer in Iran (3, 5). Because of the similarity of symptoms of gastric cancer to other diseases of the gastric, in most cases this cancer is diagnosed at advanced stage, so that survival of patients would be low (3). Many studies have been carried out on gastric cancer, survival analysis of the patients and the identification of risk factors of this disease. But most of the statistical models used in them, such as Cox proportional hazards model and parametric models, make assumptions such as establishing normal distribution for response variable, the linear relationship between independent variables and response variable, similarity of errors, etc. for data distribution (8-6), while these assumptions are not applicable in many cases. Artificial neural network models do not consider any assumption for distribution of data, and they could model complex nonlinear relationships and high-grade interactive effects based on internal relationships, without prejudices about any form of distribution (9, 10). In this method there is also the possibility that the malfunctioning of a part of neurons would not cause the complete breakdown of the network, and yet it would be likely to make right decisions (11). In addition, interoperability of the model allows providing an appropriate response to patient's situation based on the new condition risk (12). This study was carried out to estimate the survival rate of patients with gastric cancer and determine the influential factors by using artificial neural network. 

## Methods

This is a historical cohort study. A total number of 430 patients with gastric cancer who referred to Baghban clinic in Sari, from early November 2006 to late October 2013 was studied. Patients diagnosed with the disease by the physicians and those with less than 50% of available information were excluded. Patient's information during and after the conduct of the investigation is kept confidential and is not available to others. Survival period was considered as the dependent variable. Also age, gender, body mass index (BMI), high risk dietary habits (including consuming high-calorie foods, salty and smoked foods, low amounts of fruit and vegetables, frozen meals, high amounts of salt and drinking hot tea, all of which yes or no), family history (yes or no), history of chronic diseases (yes or no), history of smoking and alcohol (yes or no), occupation (including gardener, farmer, miner (coal), one who works with toxic spills, housewife etc.), disease histology (wound, ulcer etc.), histology type (including adenocarcinoma etc.), grade of histopathology differentiation (moderate and good), tumor stage (including early stages, localized enlargement, distant metastasis), tumor size (less or more than 5cm), tumor location (Cardia, Fundus, Stomach, Antrum, Greater curvature, Lesser curvature, more than one site), type of treatment (surgery with radiotherapy and chemotherapy, radiotherapy and chemotherapy, chemotherapy, without treatment) and exposure to chemicals (yes or no) were examined as independent variables. All of these variables are extracted from medical records and the last health status of patients got to be known through phone calls and were recorded in the provided check lists, and patients’ survival time were calculated in terms of months.

Data was analyzed by using SPSS v.18.0 and the level of significance was considered α=0.05. Missing observations were estimated using regression method. Then, Cox proportional hazards model and Kaplan-Meier nonparametric methods were used for data analysis. Comparison of survival rates was made by log-rank test. The final analysis of data was performed by artificial neural network pattern on significant variables.

Artificial neural networks are computational tool inspired by the human brain and are a part of dynamical systems that transfer knowledge or rules concealed in the data to the network structure by processing the experimental data. Neuron is the smallest unit of information processing that form the basis of neural network performance. All artificial neural networks are divided into two categories: supervised and unsupervised learning systems.

Learning systems are those systems that could present appropriate behaviors depending on conditions according to the available models, and could improve their performance in order to achieve a specific purpose only through observing system’s operation. The system starts the process by the random selection of initial weights, and then continues the process of training and learning.

A neural network normally has three layers: input, intermediate (hidden) and output. All of the input layers information are transferred to the output layer in a layered way. Input layers could be output for the other layer or as raw data in the first layer in the form of numerical data, literary texts, images etc.

The main task of the middle layer is to extract classified information from the existing data. Also the output layer shows the final output of the network. For analyzing with this method, firstly data were randomly divided into two parts: training and testing. What is important in neural networks is the proper choice of weights and bias sections of the network if needed. Choosing the weights is known as learning algorithms and is regarded as a key part of network distinctions in the methodologies of their parameter setting (13). To fit neural network model, first, censored patients were separated and non-censored patients were divided into two groups, 211 for training and 72 for validation group. To ensure that there is no significant difference in the distribution of independent variables between the two groups, chi-square statistics were used, and no significant difference was found between the two sets of data for the distribution of independent variables.

The observed survival rates of the two groups were tested by log rank test and no significant difference was shown between the median survivals. For fitness of the ANN model, a three-layer of neural network including 17 input nodes, three hidden nodes and two output nodes were selected as architecture of the network. Since the output of the network, i.e. the status of each patient, is a binary variable, we applied sigmoid function as the activation function of the output layer. By using data sets of training and supervised back-propagation algorithm of learning, neural network was trained; and the training process was stopped when no reduction was made in the error of the test group. Also the sigmoid function was considered as activation function of the hidden layer. The mean square error and Receiver Operating Characteristic (ROC) curve were used as indicators for determining the best network.

## Results

Among the 430 patients with gastric cancer, 296 (68.6%) cases were male and 134 (31.4%) were female, so the proportion of male to female was 2/2. The total average age of the studied patients was (64.45 ±13.56) years (65.98 ±12.22 for male and 61.12 ±15.66 for female). 9% of the patients were under the age of 45 and 56% were over 65 years.

Based on Job information available for 258 patients, 100 men were farmers and stockmen and 95 women were housewives. 52.1% of patients lived in urban areas and 47.9 % in rural areas. 36% of patients were smokers and 7% of them had a history of consuming alcohol. Also in the case of dietary habits, 24% of the patients had salt intake, 59.1% had a high consumption of hot tea, 12.9% had frozen meals in their diet, 14.3% consumed high-calorie foods, 5% had salty and smoked food in their diet, and 33.7 % consumed very small amounts of fruit and vegetables in their diets. The location of the tumor in 64 cases (24.8%) of patients was in the cardiac and for 62 patients (24%) was in the gastric antrum. According to the available information of the type of tumor for 179 patients, in 79.9% of cases the tumor has appeared as a scar and ulcer. Among 75 patients whose tumor size was recorded in their pathology sheet, 38 patients had tumors larger than 5 cm. Also in 286 patients whose disease progression was mentioned in their records, 214 patients (74.8%) were diagnosed in the advanced stage of disease (stages 3 and 4). Patients were studied in terms of their symptoms on diagnosis and earlier, and the results are presented in Table 1.

1 to 5-year survival rates for the patients were 0.64, 0.44, 0.34, 0.28 and 0.19, respectively.

First, in univariate analyzes in order to compare survival rates in sub-groups of the estimated variables and to identify affecting factors on survival time, log-rank test was used. Variables such as the age of cancer diagnosis (p <0.001), degree of tumor differentiation (p =0.031), metastatic disease (p <0.001), stage of disease progression (p <0.001), histology type (p =0.016), the type of treatment for patients (p <0.001) and their residence (p <0.001) were found to have significant relationship with patients survival.

**Table 1:** prevalence of symptoms before diagnosis in patients

Symptoms	number	Frequency distribution	
		have	Not having
gastric ache	281	161(57.3)	120(42.7)
Reflux	281	30(10.7)	251(89.3)
Weight Loss	281	57(20.3)	224(79.7)
Loss of appetite	281	43(15.3)	238(85.7)
Dysphagia	280	40(14.3)	240(85.7)
Nausea and vomiting	280	81(28.9)	199(71.1)
Constipation	280	20(7.1)	260(92.9)
Lethargy	280	29(10.4)	251(89.6)
Anemia	282	24(8.5)	258(91.5)
Gastric bleeding	280	25(8.9)	255(91.1)

**Table 2:** Comparison of gastric cancer risk factors between training group and testing group

Variables	n=283	Training group (n=211)	Test group (n=72)	p-value
Gender				0.641
Female	88	64	24	
Male(BL)	195	147	48	
Type of histopathology				0.392
Adenocarcinoma	229	166	63	
Others	10	6	4	
Grade				0.425
Weak	26	21	5	
Middle or good(BL)	115	84	31	
Advanced stage of disease				0.63
Initial stage	49	34	15	
Local metastasis	52	39	13	
Distant metastasis	101	78	23	
Distant metastasis				0.467
Yes	118	88	30	
No	62	50	12	
Metastasis organ				0.944
Liver	74	55	19	
Others	44	33	11	
Type of treatment				0.618
Treatment1	180	136	44	
Treatment2	18	11	7	
Treatment3	61	46	15	
Treatment4	24	18	6	
Age of diagnosis				0.177
70>	165	118	47	
≥ 70	118	93	25	
Status				0.982
Death	208	155	53	
Censor	75	56	19	

**Table 3:** Percentage of independent variables importance

Examined Variables	importance	Standardized importance
Gender	0.022	7.8
The disease differentiation	0.052	18.1
Stage of disease progression	0.265	93.3
metastasis Member	0.061	21.5
Type of treatment	0.284	100
Age at time of diagnosis	0.045	16

**Figure 1:**
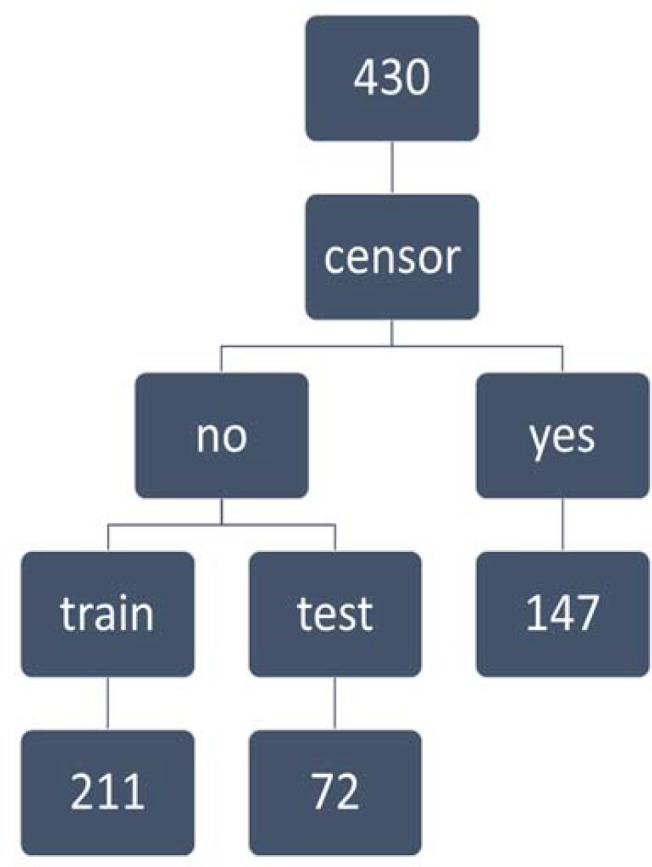
Flowchart of classification of gastric cancer patients

**Figure 2.**
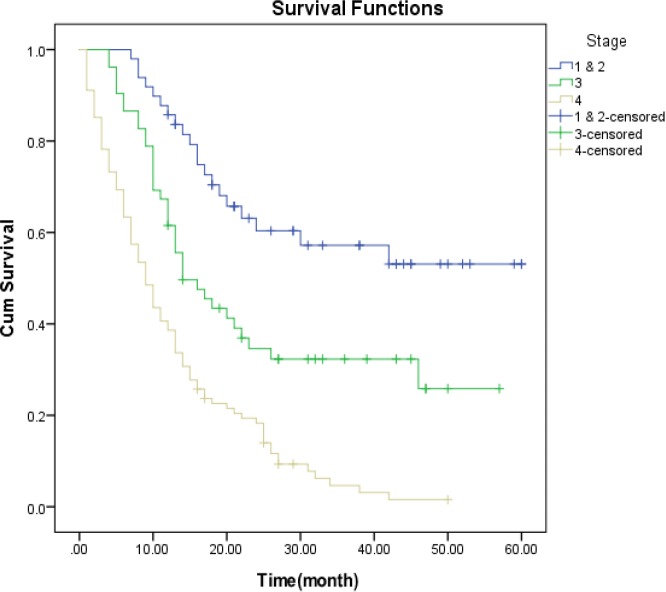
Kaplan-Meier survival estimates of gastric cancer patients by Stage of disease progression

**Figure 3:**
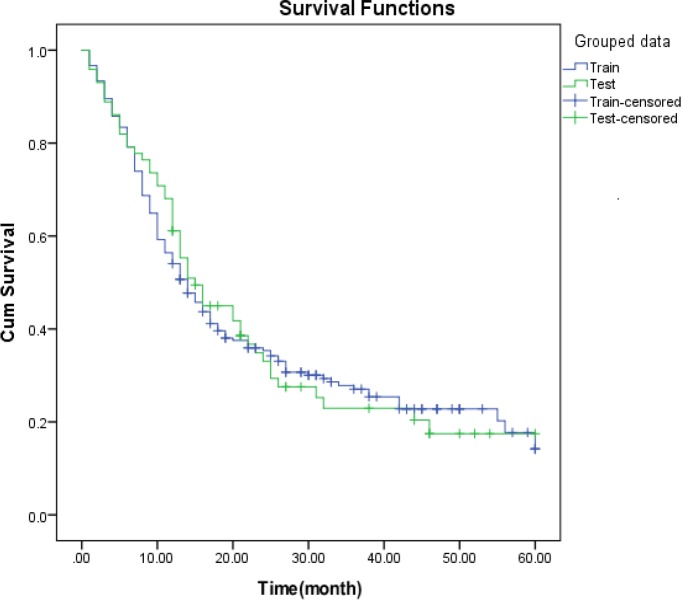
Kaplan-Meier estimate for 5-year survival rate for train and test groups

Survival curves for the stages of disease shows that the probability of survival in more patients with older age, weaker histology grade, metastases at diagnosis, advanced stages of cancer, rural residence, adenocarcinoma histology type and patients who their first treatment was a non-surgical procedure, had lower survival. 

Survival curves for the stages of disease show that the probability of survival period in advanced stages of disease have been steeper than the early stages (Figure 1).

Patients of older ages, weaker histology grade, metastases at diagnosis, advanced stages of cancer, rural residence, adenocarcinoma histology type and those whose first treatment was a non-surgical procedure, had lower survival periods. There was no significant relationship between the survival time and factors such as gender, body mass index, patient's occupation, history of smoking, history of alcohol use, history of cancer in relatives, history of chronic gastrointestinal disease, marital status, consumption of high-calorie, salted and smoked meals, low intake of fruits and vegetables, tumor location, tumor type, tumor size and type of organ metastasis. Similarly, no statistically significant relevance was found between survival time and the consumption of too much salt (p =0.077) and hot tea (p =0.178) and frozen meals (p =0.071), but they found to be closely related.

Before using a set of training and testing, chi-square statistics were used to ensure that there is no significant difference in the distribution of independent variables in the two groups, and with respect to its probability (p =0.929) no significant distinction was observed. The results of this comparison is shown in Table 2. The survival rate in the two groups was tested by log rank test and the result was not significant for the median survival of groups.

The Kaplan - Mayer graph was drawn up for the observed survival of all patients in the groups, which confirms the mentioned claim (Figure 2). 

To fit ANN model, a three-layer neural network including 17 input nodes, three- hidden nodes and two-output nodes were chosen as network architecture. Since the output of the network, i.e. the status of each patient, is a binary variable, sigmoid function was used as an activation function of the output layer.

Considering the importance of the independent variables, treatment variable with standard of 100% and then the stage of disease progression with 93.3% and the age with 16% were found as the most important and least important variables, respectively (Table 3). The percentage of the correct predictions of the present network and ROC curve were 92%, 94%, respectively.

## Discussion

In the present study 296 (68.6%) of the studied patients are male and 134 (31.4%) are female, thus the gender ratio is 2.2, that corresponds to the similar studies conducted in Ardabil, Fars and Tehran (14, 15, 16). 

This study found the most age prevalence to be in the seventh decade of life and the results of other studies confirm our findings (17, 18). The total average age of patients was about 64.45 years (65.98 for male and 61.12 for female) that is higher than the average age estimation in other studies (15, 19-21). 

The results of log rank test showed that there is a significant distinction between the longevity of patients and the cancer progression stage, so that patients with stage IV had the least survival; this result is consistent with the study conducted by Moghimi Dehkordi, Biglarian, and Yazdani in Mazandaran province and Khedmat (15, 20, 22, 23). In this study, 1 to 5 years survival rates for patients were 0.64, 0.44, 0.34, 0.28 and 0.19, respectively, that matched with Zeraati’s studies in Tehran (three-year survival of 0.31 and the five-year survival of 0.18) and Biglarian in Tehran (three-year survival of 0.32), while did not match with the study of Ismaili in Mazandaran province and Yazdanbod in Ardebil (20, 22, 24, 25). The five-year survival rate has been reported in developed countries including America 0.37, Switzerland 0.22, France 0.30, China 0.30, and Japan 0.35 in 1992 and 0.89 in 2003 (26, 28). The low survival rate of patients could be due to their late referral and delay in diagnosis, because of the similarity of symptoms among gastric diseases.

In this research, family history was not found to be an effective variable and this finding is consistent with the results of Moghimi Dehkordi, Yazdanbod and Biglarian, but does not match with the studies in other countries (14, 15, 29). As expected, histology grade variable in this study is known as an influential factor on survival in gastric cancer. This means that patients who are diagnosed with well-differentiated grade level have lower risk of death and this is confirmed by studies in Japan and Spain (24, 25). A study by Pourhoseingholi and colleagues in Iran also showes that patients with a lower degree of differentiation encounter a higher risk of death (21). Presence or absence of metastasis is significantly associated with survival time that is confirmed by studies in other countries and is consistent with the results of studies carried out by Moghimi Dehkordi and Biglarian (15, 29).

In this research, treatment was recognized as a factor affecting survival of patients, so patients who have used surgical treatment with chemotherapy and radiotherapy have a higher survival rate than patients who had surgery with chemotherapy. Also patients who have been treated surgically with chemotherapy have higher survival rate than patients who were treated just surgically.

Studies in North America, China and Europe have proved the complementary treatment effect of chemotherapy and radiotherapy on patients’ survival (26, 28).

In 2007, the American Cancer Research Association announced that the consumption of certain meals could increase the risk of incidence and development of the disease (30). A study on the immigrant population has also emphasized the role of dietary factors as one of the most important causes of gastric cancer. Some epidemiological, case-control, and cohort studies suggest that the risk of this cancer is increased with the consumption of highly salted meals, salted and processed meat, and decreases with the high consumption of fruits and vegetables (31, 32). In this study, 24% of patients had a history of salt intake, 59.1% had a high consumption of hot tea, 12.9%, used to eat frozen meals, 14.3% were accustomed to use high-quality meals, 5% profitable and smoked foods, and 33.7 percent have been taking very small amounts of fruits and vegetables.

In this research, the age of cancer diagnosis also found to be an influential factor of survival and patients whose disease is diagnosed at a younger age, have higher survival than others. This may be due to the lower progression of disease or the better physical condition at younger ages. These results are consistent with studies done by Yazdani, Pourhoseingholi, Moghimi Dehkordi and other projects conducted in other countries, but is contradicted with a study conducted in Mazandaran province (15, 22, 25, 33). Some missing information in patients’ records, incomplete pathology reports and the failure in registering certain important information such as the progression of the disease, histology grade and tumor size in patient's files, as well as not having the access to patients or their families due to changes in contact information were some limitations of this study. Using precise statistical method for predicting patient survival and identifying related factors could be considered as the strengths of this study.

In this study, artificial neural network model was used to predict the survival of patients with gastric cancer and the results showed that treatment type with standard of 100% and the disease progression stage with 93.3% were the most important independent variables, and age with 16%, was of the least importance.

Distant metastasis and disease progression variables have been removed from the final output because of the less importance of network. Finally, based on the study results, we found that the 5-year survival rate of patients with gastric cancer in Sari is low, the reason of which could be the delays in diagnosis and referral. Therefore, the use of screening methods and early diagnosis could be influential for improving survival of these patients.
